# Estimation of basal metabolic rate in Chinese: are the current prediction equations applicable?

**DOI:** 10.1186/s12937-016-0197-2

**Published:** 2016-08-31

**Authors:** Stefan G. Camps, Nan Xin Wang, Wei Shuan Kimberly Tan, C. Jeyakumar Henry

**Affiliations:** 1 Clinical Nutrition Research Centre (CNRC), Singapore Institute for Clinical Sciences (SICS), Agency for Science, Technology and Research (A*STAR), 30 Medical Drive, Singapore, 117609 Singapore; 2 Department of Biochemistry, Yong Loo Lin School of Medicine, National University of Singapore, Singapore, Singapore

## Abstract

**Background:**

Measurement of basal metabolic rate (BMR) is suggested as a tool to estimate energy requirements. Therefore, BMR prediction equations have been developed in multiple populations because indirect calorimetry is not always feasible. However, there is a paucity of data on BMR measured in overweight and obese adults living in Asia and equations developed for this group of interest. The aim of this study was to develop a new BMR prediction equation for Chinese adults applicable for a large BMI range and compare it with commonly used prediction equations.

**Methods:**

Subjects were 121 men and 111 women (age: 21–67 years, BMI: 16–41 kg/m^2^). Height, weight, and BMR were measured. Continuous open-circuit indirect calorimetry using a ventilated hood system for 30 min was used to measure BMR. A regression equation was derived using stepwise regression and accuracy was compared to 6 existing equations (Harris-Benedict, Henry, Liu, Yang, Owen and Mifflin). Additionally, the newly derived equation was cross-validated in a separate group of 70 Chinese subjects (26 men and 44 women, age: 21–69 years, BMI: 17–39 kg/m^2^).

**Results:**

The equation developed from our data was: BMR (kJ/d) = 52.6 x weight (kg) + 828 x gender + 1960 (women = 0, men = 1; R^2^ = 0.81). The accuracy rate (within 10 % accurate) was 78 % which compared well to Owen (70 %), Henry (67 %), Mifflin (67 %), Liu (58 %), Harris-Benedict (45 %) and Yang (37 %) for the whole range of BMI. For a BMI greater than 23, the Singapore equation reached an accuracy rate of 76 %. Cross-validation proved an accuracy rate of 80 %.

**Conclusions:**

To date, the newly developed Singapore equation is the most accurate BMR prediction equation in Chinese and is applicable for use in a large BMI range including those overweight and obese.

## Introduction

Since the classical experiments by Lavoisier and Laplace in 1783, energy metabolism remains the central tenant of human nutrition. Basal metabolic rate (BMR) was introduced to describe energy expended at rest in contrast to energy expended during physical activity and has been described as the “minimal rate of energy expenditure compatible with life” [1]. It represents the energy required for maintenance, necessary to sustain and maintain the integrity of vital functions and is mainly determined by the amount of lean tissue. Generally, BMR represents 60–80 % of total daily energy expenditure (TDEE) [2, 3].

In the 1985 FAO/WHO/UNU report on human energy and protein requirements, the use of energy expenditure rather than food intake was proposed to calculate energy requirements and additionally, it was proposed that TDEE can be expressed as multiples of BMR, defined as physical activity level (PAL) [3–7]. This approach to estimate energy requirements necessitates the accurate estimation of BMR in populations of different ethnicities and body weight and living under various environmental conditions.

Indirect calorimetry, which is based on the measurement of oxygen consumption and carbon dioxide production, is the preferred method to accurately assess BMR. It requires subjects to be at rest while being awake, fasted for at least 10 h, in a supine position, and under thermoneutral conditions [8]. Measurement equipment can be costly, is not widely available, requires trained personnel, and can be time consuming. Therefore, indirect calorimetry is not feasible in daily practice. Consequently, predictive equations to estimate energy requirements are the order of the day in dietetic practice and for most clinical and inpatient care. Commonly, the prediction equations are based on readily available physical measures such as age, sex, height and weight [9].

Among the first widely used prediction equations were the equations developed by Harris and Benedict (HB) in 1918 and the FAO/WHO/UNU recommended prediction equations based on the 1985 Schofield database [10, 11]. Over the years, several studies have shown that these equations overestimate BMR in tropical populations [12–17] because in tropical populations BMR is 15–20 % lower compared to Europeans and Americans [18–20]. This explains the overestimation by the Schofield and HB equations because respectively 87 % and 100 % of the data came from Caucasian men and women. Likewise, it has been shown that these equations overestimate BMR in overweight and obese populations [21–23].

Consequently, improved equations have been developed in an attempt to improve estimates of BMR and reflect racial variations [12, 14, 24–29]. Among these are the EU recommended Henry equations, and the equations of Liu et al. and Yang et al. [8, 25, 26], which have in common that they have been developed in predominantly healthy Asian subjects. Similarly, Mifflin et al. and Owen et al. have reported improved predictive equations for overweight and obese Caucasian subjects [30–32]. However, there is a paucity of data on BMR measured in overweight and obese adults living in Asia and equations developed for this group is of interest.

Since the prevalence of overweight and obesity has been rising globally since the 1980s with no exception in Asia, there is an increased need for accurate assessment of daily energy requirements to plan for healthy weight management or appropriate weight loss [33–35]. Current prediction equations for BMR to estimate energy requirements have not been developed specifically for the overweight and obese Chinese population.

Therefore, the first objective of this study was to develop BMR predictive equations in Chinese adults applicable for a large BMI range. The second aim was to compare the accuracy of the newly developed equations with a) commonly used prediction equation: Harris-Benedict equation, b) prediction equations suitable for a broader ethnic range: Henry equations, Liu equation and Yang equation and c) prediction equations advised for use in overweight and obese subjects: Mifflin and Owen equation. Thirdly, the newly derived prediction equation was cross-validated in a separate group of Chinese subjects to confirm the applicability.

## Subjects and methods

### Subjects

A total of 232 Singaporean Chinese subjects (121 men and 111 women, age: 21–67 years, BMI: 16–41 kg/m^2^) participated in the cross-sectional study. These were used to generate the regression equation. A separate set of 70 Singaporean Chinese subjects (26 men and 44 women, age: 21–69 years, BMI: 17–39 kg/m^2^) was used to cross-validate the new equation. They underwent a pre-participation screening that included the completion of a questionnaire on general health. Subjects were of Chinese descent, in good health, not using medication (except for contraception), were non-smokers and at most moderate alcohol consumers. They were weight stable as defined by a weight change <3 kg for at least 6 months prior to the study. The study was conducted according to the guidelines laid down in the Declaration of Helsinki and all procedures were approved by the National Healthcare Group Domain-Specific Review Board (2013/00783). Written informed consent was obtained from all participants. This trial was registered at www.anzctr.org.au as ACTRN12614000643673.

### Study design

Subjects were required to undergo a 12 h overnight fast and refrain from intensive physical activity for 24 h prior to the measurement. On the test day, subjects reported to the Clinical Nutrition Research Centre at 0830 h. They were instructed to travel by public transport or by car and use the elevator to avoid physical activity that would increase BMR. Subjects were then allowed to lie supine quietly and relax for 15 min before measurement of BMR via indirect calorimetry commenced. Subsequently, anthropometric measurements were performed. All measurements were performed by two researchers experienced in the field of nutrition and energetics.

### Basal metabolic rate

BMR was measured (BMRm) continuously by open-circuit indirect calorimetry using a ventilated hood system (Quark CPET, COSMED, Rome, Italy) for 30 min. For this, a transparent Perspex ventilated hood was placed over the subjects head, through which outside air was drawn by a pump. The flow rate (20–40 L/min) was measured and adjusted to keep the difference in carbon dioxide readings between inspired and expired air within the range of 0.8–1.2 %. A small sample of air leaving the hood was analysed for oxygen (O_2_) and carbon dioxide (CO_2_) by a paramagnetic analyser and infrared analyser respectively. Calibration of flow and gas analysers were calibrated with dried standard gas mixture (16.01 % O_2_, 4.98 % CO_2_) and dried atmospheric air (20.93 % O_2_, 0.03 % CO_2_). The validity of the ventilated hood system was further tested by ethanol combustion tests conducted biweekly. All measurements were carried out in a quiet room with an ambient temperature between 23–25 degrees Celsius, barometric pressure of 750–770 mmHg and constant humidity of 60 %. During the measurements, subjects were lying in a semi-supine position, quiet, motionless and were kept awake. O_2_ and CO_2_ concentrations of the outflowing air and the airflow rate through the hood were measured every 5-s to obtain oxygen consumption and carbon dioxide production. To eliminate effects of subject habituation to the test procedure, the respiratory measurements during the first 5 min were discarded. Only steady-state periods of measurements of 10 min were used to calculate BMR. BMR was calculated from oxygen consumption and carbon dioxide production by the Weir formula [36]. In addition to measured values, BMR was predicted (BMRp) by the Harris-Benedict, Henry, Liu, Yang, Owen and Mifflin equations (Table 1) [8, 11, 25, 26, 30–32].

**Table 1:** Prediction equations to calculate BMR (kJ/d)

Equations	Age	Men	Women
Singapore	Adult	52.6 W + 2788	52.6 W + 1960
HB [11]	Adult	278 + 57.5 W + 20.9H– 28.2A	2783 + 40.0 W + 7.7H– 19.5A
Henry [8]	18–30	60.0 W + 13.1H + 473	43.3 W + 25.7H – 1180
	30–60	47.6 W + 22.6H – 574	34.2 W + 21.0H – 49
	60–70	47.8 W + 22.6H – 1070	35.6 W + 17.6H + 45
Liu [26]	Adult	58.1 W + 17.4H – 14.4A + 227	58.1 W + 17.4H – 14.4A – 243
Yang [25]	Adult	89 W + 877	89 W + 277
Mifflin [30]	Adult	41.8 W + 26.2H -20.6A +21	41.8 W + 26.2H -20.6A – 674
Owen [31, 32]	Adult	42.7 W + 3678	30.0 W + 3326

*W* weight (kg), *G* gender (men = 1, women = 0), *H* height (cm), *A* age (years), *HB* Harris and Benedict

### Anthropometric measurements and body composition

Height was measured to the nearest 0.1 cm using a calibrated electronic weighing and measuring station (Seca 763, Medical Scales and Measuring System, Hamburg, Germany) and weight to the nearest 0.001 kg using a calibrated scale (Life Measurement Corporation, Inc, Concord, CA, USA). Both height and weight measurements were taken twice, and the mean of the two measurements was recorded. BMI was calculated as weight (kg) divided by height (m) squared. The conventional cut-off point of BMI greater than 25.0 to describe overweight and obesity in Caucasians does not apply in Asians. Hence, in this study a BMI greater than 23.0 will be used to define overweight and greater than 27.5 to define obesity [37].

### Statistical analysis

Statistical analysis was performed using Microsoft Excel 2010. Statistical significance was set at *P* < 0.05 (two-sided test). All results were expressed as mean ± standard deviation. Multiple stepwise regression analysis was used to derive the new prediction equation (Singapore equation) to estimate BMR based on weight, height, age and gender as independent variables. Pearson’s coefficient of determination (R^2^) and the residual standard deviation (RD) were calculated as measures of goodness of fit between the measured and predicted regression equations used. The degree of agreement between the predicted and measured BMR was evaluated by Bland-Altman limits of agreement analysis [38]. The limits of agreement were defined as the mean difference ±2 standard deviations. The estimated accuracy was defined as the percentage of the subjects whose BMRp was within ±10 % of BMRm. Overestimation and underestimation were defined as >10 % and <10 % of BMRm, respectively [21]. Estimated accuracy was used to cross-validate the newly derived equation in an independent group of 70 subjects. Pearson’s coefficient of determination was calculated as measure of goodness of fit between the BMRm and BMRp from the newly derived equation.

## Results

**Fig. 1:**
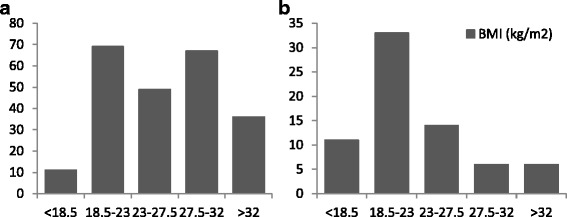
Subject distribution according to body mass index (BMI) of **a** cross-sectional study (*n* = 232) and **b** cross-validation study (*n* = 70)

Subject characteristics for the separate cross-sectional study and cross-validation study are summarised in Table 2a and b and BMI distribution is shown in Fig. 1. There were no significant differences between men and women for age and BMI.

**Table 2:** Subject characteristics (mean ± SD)

a.			
cross-sectional study	Men (*n* = 121)	Women (*n* = 111)	Total (*n* = 232) (range)
Age (years)	32.3 ± 9.9	33.4 ± 11.2	32.8 ± 10.5 (21.6–66.8)
Weight (kg)	79.2 ± 14.9	66.1 ± 16.1	72.9 ± 16.8 (39.3–113.1)
Height (cm)	171.7 ± 5.9	159.9 ± 6.2	166.1 ± 8.5 (147.4–189.3)
BMI (kg/m^2^)	26.9 ± 4.9	25.8 ± 5.9	26.4 ± 5.4 (16.4–40.8)
BMR (kJ/d)	6958 ± 1033	5439 ± 908	6230 ± 1234 (3615–10213)
b.			
cross-validation study	Men (*n* = 26)	Women (*n* = 44)	Total (*n* = 70) (range)
Age (years)	28.2 ± 6.3	28.8 ± 10.2	28.6 ± 10.45 (21.6–66.8)
Weight (kg)	72.1 ± 13.3	59.7 ± 15.6	64.3 ± 15.8 (40.7–104.6)
Height (cm)	173.4 ± 6.3	162.3 ± 5.3	166.4 ± 7.8 (151.9–185.5)
BMI (kg/m^2^)	24.0 ± 3.9	22.7 ± 5.8	23.2 ± 5.2 (16.6–38.9)
BMR (kJ/d)	6473 ± 941	5075 ± 870	5594 ± 1121 (3774–8301)

*BMI* body mass index, *BMR* basal metabolic rate

Multiple stepwise regression analysis was used to determine which variables independently contributed to the prediction of BMR. Weight, height and gender were significantly correlated to BMR; however height was excluded following stepwise regression analysis resulting in the following BMR prediction equation: BMR (kJ/d) = 52.6 x weight (kg) + 828 x gender + 1960 (women = 0, men = 1) + 468.36 (*R* ^2^ = 0.81, *P* < 0.001) (Table 1). Inclusion of weight resulted in a RD of 656 (kJ/d) and inclusion of gender improved RD to 534 (kJ/d); inclusion of either or both height and age did not improve RD.

**Fig. 2:**
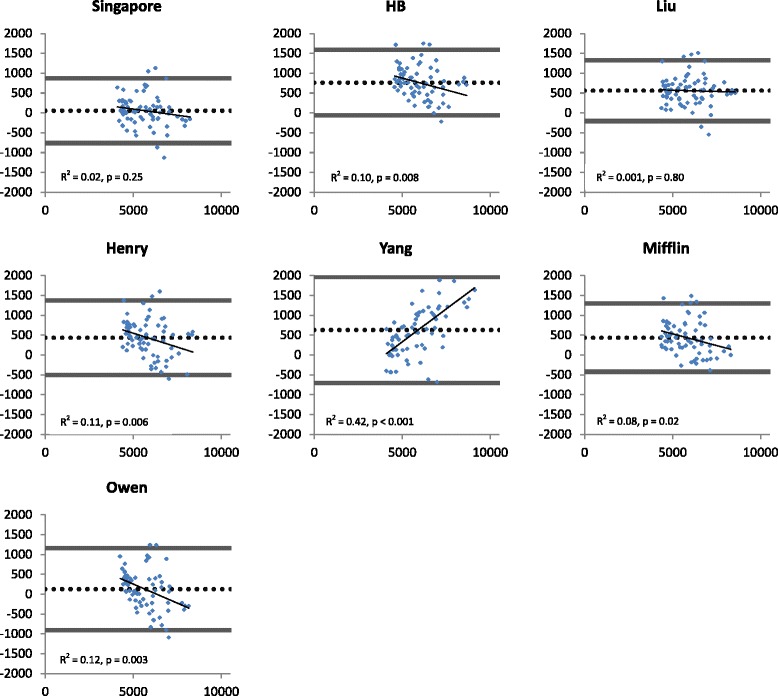
Bland-Altman plots describing agreement between measured BMR and predicted BMR from 7 equations in the cross-validation study (*n* = 70). Delta BMRp – BMRm (kJ/d) is plotted against the average of BMRm and BMRp (kJ/d). Reference lines represent the mean error (dotted line) of the prediction equation (bias) and the limits of agreement (±2 SD) (solid line). Regression lines, their coefficient of determination and *p*-value for the slope are provided. BMRp = predicted basal metabolic rate, BMRm = measured basal metabolic rate, HB = Harris and Benedict

Predicted BMR values in the cross-sectional and cross-validation study by all prediction equations and the differences with measured BMR values are summarised in Table 3a and b. The second column shows the mean difference between BMRm and BMRp with a positive value being an overestimation by the prediction equation. Paired t-tests were used to compare the differences between BMRm in both studies and BMRp using the prediction equations. This was not applicable for the predicted BMR by the Singapore equation in the cross-sectional study as by design of the study. The validation study showed that only BMRp by the Singapore equation showed no significant difference from BMRm. Significant correlations were found between BMRm and BMRp for all equations. For the cross-validation study, the individual differences between measured and predicted BMR plotted against the average of the measured BMR and predicted BMR are shown in Fig. 2. The limits of agreement were wide for all equations while only the Singapore and Liu’s equations did not show directional bias.

**Table 3:** Mean predicted BMR and difference with measured BMR

	Mean ± SD (kJ/d)	Bias ± SD (kJ/d)	P value in paired t-test	R^2^	Limits of Agreement (kJ/d)
a.					
Cross-sectional study (*n* = 232)					
Singapore	6230 ± 1115	-3 ± 534	NA	0.81*	-1070 to 1064
HB [11]	6777 ± 1124	545 ± 607	<0.0001	0.76*	-669 to 1759
Henry [8]	6362 ± 1003	131 ± 655	<0.005	0.72*	-1179 to1440
Liu [26]	6558 ± 1187	426 ± 745	<0.0001	0.79*	-718 to 1570
Mifflin [30]	6397 ± 1080	165 ± 621	<0.0001	0.74*	-1076 to 1406
Owen [31, 32]	6214 ± 1036	-17 ± 604	0.66	0.79*	-1225 to 1190
Yang [25]	7028 ± 1638	850 ± 786	<0.0001	0.79*	-721 to 2421
b.					
Cross-validation study (*n* = 70)					
Singapore	5651 ± 1055	56 ± 407	0.25	0.87*	-759 to 870
HB [11]	6356 ± 998	761 ± 413	<0.0001	0.87*	-65 to 1586
Henry [8]	6029 ± 980	434 ± 469	<0.0001	0.83*	-503 to 1371
Liu [26]	6154 ± 1107	559 ± 381	<0.0001	0.89*	-204 to 1322
Mifflin [30]	6030 ± 1000	435 ± 429	<0.0001	0.85*	-424 to 1293
Owen [31, 32]	5722 ± 934	126 ± 516	0.044	0.79*	-907 to 1159
Yang [25]	6225 ± 1545	630 ± 668	<0.0001	0.85*	-707 to 1967

*P < 0.001

*HB* Harris and Benedict

Table 4 summarises percentages of accurate (accuracy rate), underestimated and overestimated BMR predictions by all equations for all subjects of the cross-sectional and cross-validation study and subcategorized for a BMI smaller and greater than 23 for the cross-sectional study. A value is considered accurate when the difference between BMRp and BMRm is not greater than ± 10 %. In the cross-validation part of the study, the accuracy rate for the newly derived prediction equation reached 80 %, while under- and overestimation were respectively 4 % and 16 %. Goodness of fit between BMRm and BMRp from the new equation was good (*R* ^2^ = 0.87).

**Table 4:** The percentage of accurate, underestimated and overestimated BMR predictions for all subjects and subcategorized for a BMI smaller and greater than 23, BMR prediction equations are ranked according to overall accuracy for the cross-sectional study (*n* = 232) and underestimated and overestimated BMR predictions for all subjects of the cross-validation study (*n* = 70)

	Cross-sectional study									Cross-validation study		
	underestimation			accurate within ± 10 %			overestimation			under	accurate	over
	All	≤23	>23	All	≤23	>23	All	≤23	>23	All	All	All
Singapore	9 %	6 %	11 %	78 %	80 %	76 %	13 %	14 %	13 %	4 %	80 %	16 %
Owen [31, 32]	14 %	2 %	20 %	70 %	74 %	68 %	16 %	24 %	12 %	9 %	70 %	21 %
Henry [8]	9 %	2 %	13 %	67 %	59 %	72 %	24 %	39 %	16 %	2 %	59 %	41 %
Mifflin [30]	9 %	1 %	13 %	67 %	61 %	70 %	24 %	38 %	16 %	1 %	59 %	41 %
Liu [26]	3 %	0 %	5 %	58 %	63 %	56 %	38 %	37 %	39 %	0 %	49 %	51 %
HB [11]	4 %	1 %	6 %	45 %	33 %	52 %	50 %	66 %	42 %	0 %	37 %	63 %
Yang [25]	2 %	4 %	1 %	37 %	69 %	20 %	61 %	27 %	79 %	4 %	44 %	56 %

*BMR* basal metabolic rate, *BMI* body mass index (kg/m^2^), *HB* Harris and Benedict

## Discussion

Measuring basal metabolic rate in a group of Chinese adults allowed us to develop an accurate BMR prediction equation (Singapore equation) applicable for a large BMI range including overweight and obese. The equation, based on weight and gender, proved to have a high rate of accurate BMR predictions which was confirmed in a cross-validation study.

Basal metabolic rate is defined as the energy required to sustain and maintain the integrity of vital functions or the minimal rate of energy expenditure compatible with life [1]. In literature, controversy exists whether measurement conditions meet BMR requirements or provide resting metabolic rate [39]. In our study BMR was measured using indirect calorimetry under the essential standard conditions requiring subjects to be at rest while being awake, fasted for at least 10 h, in a supine position, and under thermoneutral conditions [8]. An overnight stay at the research centre before the measurement is sometimes suggested but is often not practical. The newly developed Singapore equation based on these measurements estimated BMR accurately in almost 80 % of the subjects across the wide BMI range, which was confirmed in the cross-validation population. The Singapore equation can now be used to estimate daily energy requirements in Chinese overweight and obese adults to plan for healthy weight management or appropriate weight loss.

Previously, the equations of Henry, Liu and Yang were developed to improve estimates of BMR and reflect ethnic variations. Compared to the Schofield database, the Henry equations contained a larger number of people from the tropics and they excluded the Italian subjects from the Schofield database [8]. The European Food Safety Authority recommends the use of the Henry equations to predict BMR in all 27 countries of the European Union [40]. In our Chinese study population, the Henry equations showed a small overestimation which ranked 3^rd^ lowest (131 kJ/d) and showed the 3^rd^ highest accuracy rate (67 %) on BMR predictions in the cross-sectional study and similar in the validation study. Additionally, Henry’s equations showed an improvement in accuracy rate in subjects with a BMI above 23. The equation from Liu et al. was developed in 223 healthy Chinese adults within normal limits for body weight [26] and has been recommended to predict BMR in Chinese [14–16] while Yang’s equation was developed in 165 normal weight Chinese subjects [25]. Though, in our Chinese study population both equations overestimated BMR while they were accurate in 58 % (Liu) and only 37 % (yang) of the subjects, and performed equally in the validation study. The equation by Liu et al. showed an even accuracy rate across the BMI range in contrast to Yang’s equation which decreased to 20 % for overweight and obese subjects but improved to 69 % (3^rd^ highest) in normal weight subjects; in line with their own conclusions [25]. In comparison, the Harris-Benedict equation showed an average overestimation of 545 kJ/d while the BMR prediction was accurate in 45 % of the subjects. Since, the Harris-Benedict equations is based on gender, height, weight and age and developed in only Caucasian subjects, the overestimation is to be expected [11, 22].

For overweight and obese populations Mifflin’s and Owen’s equations have been advised to be used in respectively American and Chinese [21, 22]. The Mifflin equation was developed in 498 normal and obese Americans while the Owen equation was developed from a group of Caucasian, Negro, and Oriental subjects [30–32]. Our results showed a 4^th^ lowest overestimation for Mifflin’s equation (165 kJ/d) and an average underestimation of BMR by 17 kJ/d for Owen’s equation in our Chinese population while accuracy rates were ranked: 3^rd^ highest for Mifflin’s and 2^nd^ highest for Owen’s equation. Their accuracy rates stayed the same or increased slightly in overweight and obese compared to normal weight subjects which could be explained by the inclusion of such subjects in their studies. They performed correspondingly in the validation study, where Owen’s equation had the 2^nd^ lowest overestimation and the 2^nd^ highest accuracy rate. Previously, Song et al. found an accuracy of 73 % by Owen’s equation for Chinese with a BMI range of 18.5–30 which is similar to the 70 % we found. They concluded that the equation of Owen provided a valid estimation of BMR in Singaporean Chinese men at group level, though critically claimed that the Owen equation displayed wide limits of agreement and directional bias across the range of BMR [22].

In summary, the results showed that BMR predictions by the Singapore equation as well as the equations by Owen et al., Henry et al. and Mifflin et al. had a low bias towards the measured BMR and were accurate around 70 % or as high as 80 % (Singapore equation) of the time. Furthermore, the results confirm that population specific prediction equations with respect to ethnicity and body type are necessary [12–16, 21–23].

The Singapore equation is limited to their derivation from our study population and the clinical utility can only be assessed by testing in other Chinese populations. Though, a cross-validation confirmed the accuracy and applicability for a large BMI range in an independent subject group with a similar wide BMI and age range. The Singapore equation did not show a significant directional bias, however it is assumed that the use of body composition could improve BMR estimation. However, therefore the Singapore equation is simple to use as it is solely based on body weight and gender. In addition, the equation confirmed that BMR is highly correlated with body weight [3] while the addition of other routinely available values (height and age) did not contribute significantly to the accuracy of predicting BMR. The strength of the newly developed Singapore equation lies in the direct practical and clinical use in a large BMI range including overweight and Chinese adults. It is important to note that BMR is the major component of TDEE; however, accurately estimating physical activity plays a role in estimating total daily energy expenditure and thus daily energy requirements.

## Conclusion

The newly developed Singapore equation reached the highest accuracy in predicting BMR in normal weight, overweight and obese Chinese Singaporeans when compared to other prediction equations, which was confirmed with a cross-validation in a separate study population.

The Singapore equation developed in the present study is the most appropriate for predicting BMR in Chinese and is applicable in a broad BMI range including overweight and obese. Their utility will be further enhanced if the equations are shown to be valid in Chinese living in other regions of Asia.

## Abbreviations

BMI: Body mass index
BMR: Basal metabolic rate
BMRm: Measured basal metabolic rate
BMRp: Predicted basal metabolic rate
PAL: Physical activity level
TDEE: Total daily energy expenditure
TEE: Total energy expenditure
